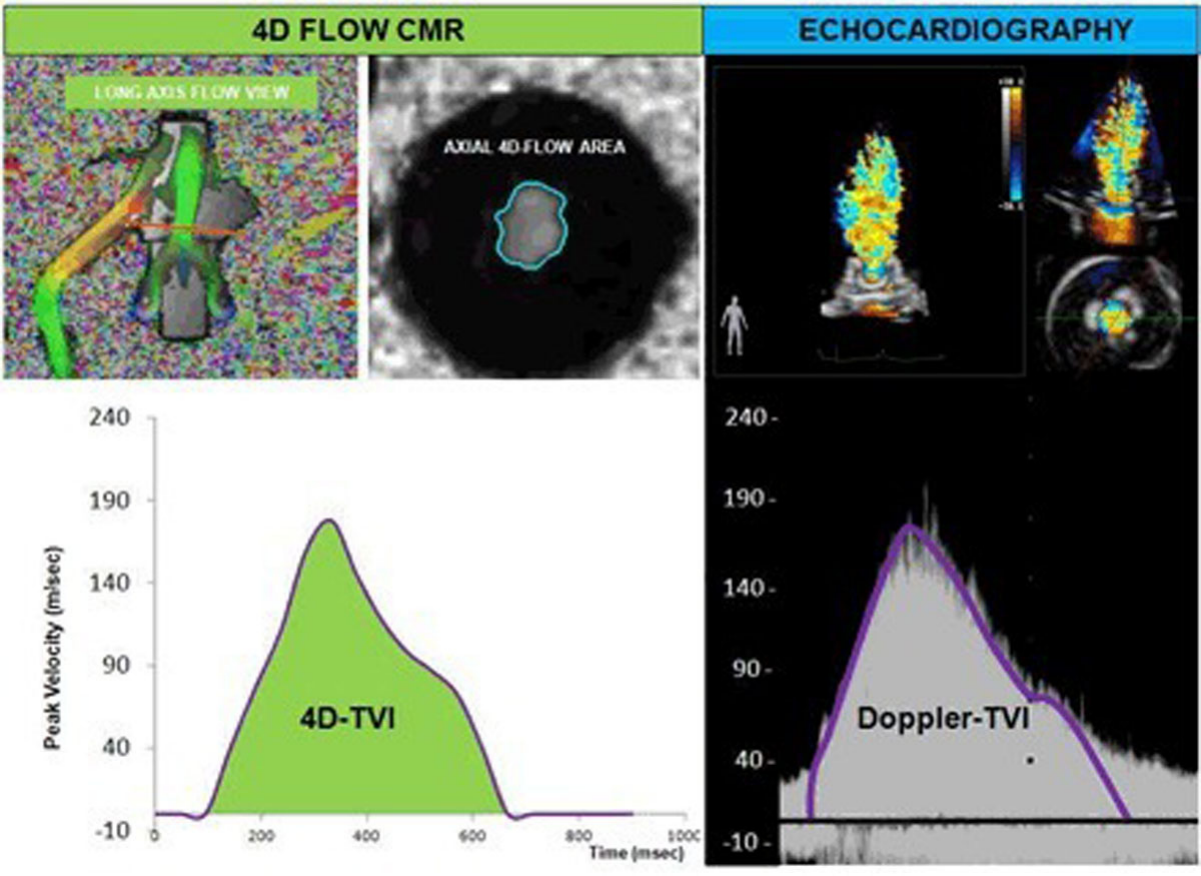# Bioprosthetic mitral valve effective orifice area using 4D flow cardiac magnetic resonance derived time velocity integral. An in vitro comparison with Doppler Echocardiography

**DOI:** 10.1186/1532-429X-17-S1-P336

**Published:** 2015-02-03

**Authors:** Dimitrios Maragiannis, Matthew Jackson, Stephen Igo, Karen Chin, Kyle Autry, Mohamad G Ghosn, Dipan J Shah, Stephen H Little

**Affiliations:** 1 Cardiology, Houston Methodist Hospital, Houston, TX, USA

## Background

4D Flow Cardiac Magnetic Resonance (CMR) is a novel imaging modality to assess bioprosthetic mitral valve (BMV) function. We describe a new, 4D Flow derived velocity time integral (TVI) based method to assess effective orifice area (EOA) for BMVs.

## Methods

In our MRI-compatible circulatory loop 4 stented porcine BMVs (27, 29, 31, 33mm) underwent CMR with a 1.5T Siemens scanner. The valves were evaluated at forward stroke volumes of 70, 90 and 110ml at a beat rate of 70bpm. We plotted instantaneous peak velocities and calculated TVI for each scenario. 4D CMR-EOA was calculated by dividing the forward stroke volume measured by our high fidelity flow transducers with the calculated TVI. Doppler derived EOA was calculated identically using the Doppler derived TVI.

## Results

4D CMR-EOA was successfully calculated in a total number of 12 tests. 4D Flow CMR-EOA revealed a strong correlation when compared with Doppler derived EOA (r=0.985, p<0.001) and the mean difference was -0.1±0.05cm^2^ between the two methods. Calculated CMR-TVI by our method correlated well with Doppler derived TVI (r=0.99, p<0.001 and mean difference 3.2±1.97cm).

## Conclusions

Our study demonstrated the feasibility of 4D Flow CMR to evaluate bioprosthetic valve EOA with the use of velocity encoded CMR data from 4D Flow Software analysis. Our data showed high agreement with Doppler EOA calculations, demonstrating a potential for 4D Flow CMR in bioprosthetic valve evaluation.

## Funding

American Heart Association grant #11BGIA5840008.

**Figure 1:**